# Supermolecule—Drug Conjugates Based on Acid-Degradable Polyrotaxanes for pH-Dependent Intracellular Release of Doxorubicin

**DOI:** 10.3390/molecules28062517

**Published:** 2023-03-09

**Authors:** Atsushi Tamura, Mamoru Osawa, Nobuhiko Yui

**Affiliations:** Department of Organic Biomaterials, Institute of Biomaterials and Bioengineering, Tokyo Medical and Dental University (TMDU), 2-3-10 Kanda-Surugadai, Chiyoda, Tokyo 101-0062, Japan

**Keywords:** polyrotaxane, cyclodextrin, doxorubicin, drug delivery system

## Abstract

Doxorubicin (DOX)-conjugated acid-degradable polyrotaxanes (PRXs) were designed as supramolecular drug carriers capable of releasing drugs in acidic cellular environments. Acid-degradable PRXs composed of α-cyclodextrin (α-CD) as a cyclic molecule, poly(ethylene glycol) (PEG) as a polymer axis, and *N*-triphenylmethyl (*N*-Trt) groups as an acid-labile stopper molecules were synthesized and DOX was conjugated with the threaded α-CDs in the PRXs. Because the acid-induced cleavage of *N*-Trt groups in PRXs leads to PRX dissociation, the DOX-modified α-CDs were released under acidic conditions (pH 5.0). The cytotoxicity of DOX-conjugated PRXs in colon-26 cells revealed significant cell death for DOX-conjugated PRXs after 48 h of treatment. Confocal laser scanning microscopy (CLSM) analysis revealed that the fluorescence signals derived from DOX-conjugated PRXs were observed in cellular nuclei after 48 h, suggesting that the DOX-modified α-CDs were released and accumulated in cellular nuclei. These results confirmed that acid-degradable PRXs can be utilized as drug carriers capable of releasing drug-modified α-CDs in acidic lysosomes and eliciting cytotoxicity. Overall, acid-degradable PRXs represent a promising supramolecular framework for the delivery and intracellular release of drug-modified α-CDs, and PRX–drug conjugates are expected to contribute to the development of pH-responsive drug carriers for cancer therapy.

## 1. Introduction

Anticancer drug delivery is a promising pharmaceutical approach in cancer chemotherapy aimed at minimizing the severe side effects of drugs while enhancing their therapeutic efficacy and enabling their preferential accumulation in target sites [1,2,3]. For example, doxorubicin (DOX), an anthracycline antibiotic isolated from *Streptomyces peucetius*, is highly active against various cancers [4,5]. However, the clinical use of DOX is limited due to its severe side effects, such as cardiotoxicity, myelosuppression, and the development of multidrug resistance [6]. To overcome these adverse effects, DOX-encapsulated liposomes, such as Doxil/Caelyx, have been developed and approved for the treatment of cancers such as Kaposi’s sarcoma, progressive ovarian cancer, and multiple myeloma [7,8]. These DOX-encapsulated liposomes contribute to attenuating the cardiotoxicity of DOX and enable its preferential accumulation in tumor tissue through enhanced permeability and retention (EPR) effects [8]. Apart from liposomal DOX, various delivery methodologies have been established for anticancer drugs, such as polymer–drug conjugates, drug-encapsulated polymer nanoparticles, and protein–drug conjugates [1,9,10]. Therefore, the establishment of new drug carriers and strategies is of significant value to the advancement of cancer chemotherapy.

Polyrotaxanes (PRXs) are representative interlocked polymers in which multiple cyclic molecules are mechanically interlocked on a linear polymer axis and the polymer terminal ends are capped with bulky stoppers [11]. Among various combinations of cyclic molecules and axle polymers, PRXs composed of α-cyclodextrin (α-CD) as the cyclic molecule, and polyethylene glycol (PEG) as the polymer axis have been extensively studied owing to the availability of facile preparation methods [12]. Recently, α-CD-based PRXs have attracted considerable interest in the field of biomaterials and drug delivery systems, owing to their unique properties, such as the molecular mobility of threading α-CDs and biocompatibility of constituent α-CD and PEG [13,14]. Moreover, threading CDs possess abundant hydroxy groups (e.g., α-CD contains 18 hydroxy groups), which are available for the conjugation of a large number of functional molecules, including drugs and imaging agents [15,16,17]. Yang et al. have conjugated anticancer drugs (e.g., DOX or camptothecin) onto threaded α-CDs in PRXs via hydrolyzable ester linkages for improving the pharmacokinetics of the conjugated drugs and achieving their sustained release in cancer cells via hydrolysis [18,19]. In another example, Wu and Jiang et al. developed paclitaxel-conjugated PRXs featuring hydrolyzable ester linkages and validated that the conjugates preferentially accumulated in tumor tissue and exhibited superior antitumor effects compared to free paclitaxel [20]. Additionally, the terminal ends of axle polymers have been utilized to conjugate anticancer drugs and other functional molecules, such as imaging agents [21,22,23]. These studies suggest that PRXs have significant potential as an emerging platform for drug delivery systems (DDSs).

Our group has proposed biocleavable PRXs containing stimuli-labile linkers at the terminal ends or center of the axle polymer to achieve the dissociation of PRXs in response to physical and chemical stimuli, such as light, pH, reductive molecules, and enzymatic reactions [24,25,26,27]. Taking advantage of the stimuli-induced dissociation of PRXs, our group has developed drug-conjugated PRXs with enzymatically labile stopper groups for the controlled release of drug-conjugated CDs [28]. Recently, our group has developed acid-degradable PRXs containing β-CDs as cyclic molecules and *N*-triphenylmethyl (*N*-Trt) groups as acid-labile stopper molecules, which are cleaved under acidic conditions, such as in lysosomes (pH 4–5), for achieving intracellular PRX dissociation and the release of threaded β-CDs [25,29]. Because β-CDs form an inclusion complex with cholesterol, acid-degradable PRXs decrease the amount of intracellular cholesterol [30]. The cholesterol-reducing ability of β-CD-threaded PRXs has resulted in superior therapeutic effects in mouse models of cholesterol-related diseases, such as Niemann–Pick type C disease and atherosclerosis, compared to those elicited by 2-hydroxypropyl β-CD [31,32,33]. Thus, acid-degradable PRXs containing *N*-Trt groups represent a judicious molecular design for realizing the dissociation of PRXs in intracellular acidic environments.

**Figure 1:**
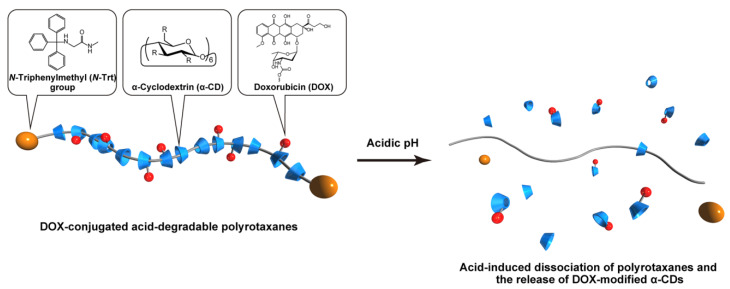
Schematic illustration of acid-degradable polyrotaxane (PRX)–drug conjugates and their acid-induced dissociation.

In this study, we aimed to apply acid-degradable PRXs as a drug carrier for achieving the intracellular release of drug-conjugated CDs through dissociation in acidic cellular environments (Figure 1). We synthesized DOX-conjugated acid-degradable PRXs comprising DOX-modified α-CDs, PEG as the polymer axis, and *N*-Trt groups as acid-labile stopper molecules (Figure 1). Because the developed PRX can be dissociated in acidic cellular environments, the threaded DOX-modified α-CDs were released in acidic lysosomes. We investigated the pH-dependent release of DOX-modified α-CDs from the PRXs, cytotoxicity of the PRX–DOX conjugates, and intracellular localization of PRX–DOX conjugates to determine the feasibility of acid-degradable PRX as a drug carrier. We believe that acid-degradable PRX–drug conjugates tested in the present study will provide new insights into the development of drug carriers for cancer chemotherapy.

## 2. Results

### 2.1. Synthesis and Characterization of DOX-HPR

**Figure 2:**
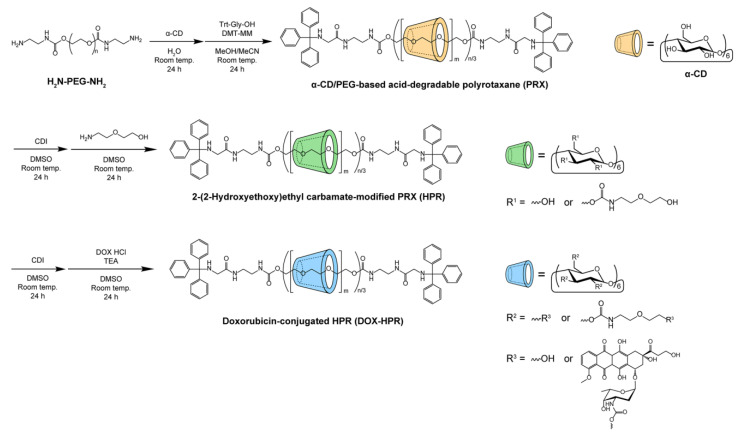
Synthetic scheme for doxorubicin (DOX)-conjugated acid-degradable PRXs.

Acid-degradable PRXs comprising α-CDs as a cyclic host molecule and PEG-bearing terminal *N*-(2-aminoethyl)carbamate groups (PEG-NH_2_; *M*_n_ = 19,800) as an axial polymer were synthesized using *N*-(triphenylmethyl)glycine (Trt-Gly-OH) as a bulky stopper molecule (Figure 2). Because protonation of the secondary amino group in *N*-Trt groups leads to the liberation of triphenylmethyl moieties [25,29], the PRX-containing *N*-Trt groups undergo pH-dependent dissociation under acidic pH conditions. From the ^1^H nuclear magnetic resonance (NMR) spectrum of the acid-degradable PRX, the number of threading α-CDs was determined to be 55.8 (Table 1), which corresponds to 26.5% threading of α-CDs in PRXs, assuming that one α-CD molecule forms an inclusion complex with two ethylene glycol repeating units [34]. Because unmodified PRX is poorly soluble in aqueous media, we incorporated hydrophilic 2-(2-hydroxyethoxy)ethyl carbamate (HEE) on the threading α-CDs in PRXs because of the excellent water solubility of HEE groups (Figure 2) [25,35]. From the ^1^H NMR spectrum of HEE-modified PRX (HPR), the number of modified HEE groups on PRX was determined to be 129, which corresponds to 2.2 HEE molecules per threading α-CD in PRX (Table 1). The resulting HPR showed high solubility in aqueous media (at least 500 mg/mL).

**Figure 3:**
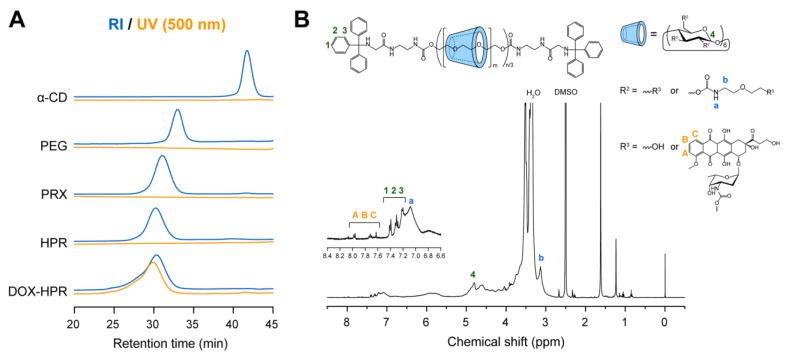
(**A**) SEC charts of α-CD, axle PEG, PRX, HPR, and DOX-HPR in dimethyl sulfoxide (DMSO) monitored with refractive index (RI) and UV (500 nm) detectors. (**B**) ^1^H NMR spectrum of DOX-PRX in DMSO-*d*_6_.

DOX was conjugated with the threaded α-CDs in HPR as shown in Figure 2; the amino groups of daunosamine sugar moieties in DOX were bonded to the hydroxy groups of threaded α-CDs or the termini of HEE groups. After the reaction, DOX-HPR was purified by gel filtration chromatography to remove any unreacted DOX. Figure 3A shows the size exclusion chromatography (SEC) charts of DOX-HPR and its precursor polymers obtained using refractive index (RI) and UV absorption (500 nm) detection. Free α-CD and DOX peaks were not observed in the SEC chart of DOX-HPR, suggesting that DOX-HPR was successfully purified. In the case of UV detection, which was performed at the maximum absorption wavelength for DOX [36], only a peak corresponding to DOX-HPR was observed. The DOX-HPR peaks detected by UV and RI appeared at similar retention times, suggesting that DOX molecules were chemically conjugated with HPR. In the ^1^H NMR spectrum of DOX-HPR, peaks assignable to the anthraquinone ring of DOX appeared at 7.63, 7.71, and 7.97 ppm (Figure 3B) [37]. In accordance with these results, we concluded that DOX-HPR was successfully prepared without contamination with free DOX. The number of DOX molecules modified onto HPR was determined to be 8.1 based on the ^1^H NMR spectrum of DOX-HPR (Table 1).

**Figure 4:**
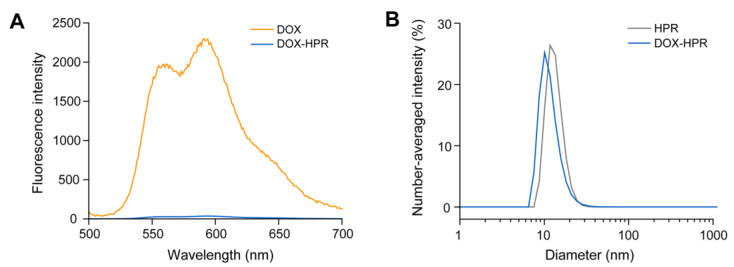
(**A**) Fluorescence spectra of DOX (1 μM) and DOX-HPR (1 μM DOX) in PBS. (**B**) Number-averaged size distribution of HPR (3 mg/mL) and DOX-HPR (3 mg/mL) in PBS.

To gain insight into the physicochemical properties of DOX-HPR in aqueous media, a fluorescence spectrum of DOX-HPR was acquired in phosphate-buffered saline (PBS). Compared with the spectrum of free DOX (1 μM), the fluorescence intensity of DOX-HPR containing an identical DOX concentration was markedly lower (Figure 4A). Because fluorescence quenching of DOX occurs due to aggregation at high concentrations [38,39,40], the data suggest that the neighboring DOX molecules conjugated on HPR were associated via hydrophobic interactions and π–π stacking [39]. These association forces of DOX often result in the formation of self-assembled nanoparticles [39]. Dynamic light scattering (DLS) measurements suggested that the size of DOX-HPR (11.8 nm) was comparable to that of HPR (13.4 nm) in PBS (Figure 4B). We considered that the self-assembly of DOX-HPR did not occur because the number of DOX conjugated on HPR was small.

### 2.2. pH-Dependent Dissociation of DOX-HPR

**Figure 5:**
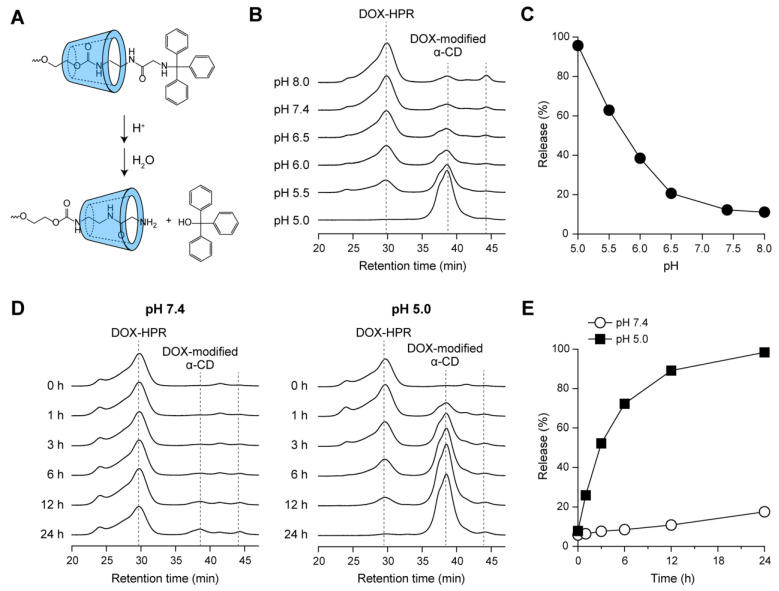
(**A**) Schematic showing the cleavage of *N*-Trt groups in acid-degradable PRXs under acidic condition. (**B**) SEC charts of DOX-HPR after incubation at pH 5.0–8.0 at 37 °C for 24 h. SEC measurements were performed using DMSO as an eluent and the peaks were detected at 500 nm. (**C**) Relationship between pH and release profiles of DOX-modified α-CDs from DOX-HPR after incubation at 37 °C for 24 h, (**D**) SEC charts of DOX-HPR after incubation at pH 7.4 and 5.0 at 37 °C for 0–24 h. (**E**) Release profiles of DOX-modified α-CDs from DOX-HPR with time after incubation at pH 7.4 and 5.0 at 37 °C.

As described above, the *N*-Trt groups used as the bulky stopper for PRX were cleaved under acidic conditions, as shown in Figure 5A [25,29]. Because the acid-induced dissociation of PRXs accompanies the release of threaded α-CDs, the DOX-modified α-CDs are expected to be released from DOX-HPR under acidic pH conditions. To gain insight into the effects of pH on the dissociation of DOX-HPR, the release of DOX-modified α-CDs from DOX-HPR was assessed using SEC after incubation at pH 5.0–8.0 for 24 h. Figure 5B shows the SEC charts of DOX-HPR obtained using UV detection at 500 nm. The peaks observed at 30.0 and 38.6 min corresponded to DOX-HPR and released DOX-modified α-CD, respectively. At neutral to mildly alkaline pH conditions, the peak intensity for the released DOX-modified α-CDs was weak, suggesting that DOX-HPR had not dissociated. However, a peak at 44.3 min was observed after incubation at neutral to mildly alkaline pH. Because DOX is hydrolyzed under neutral to alkaline conditions [40], the peak was assigned to the byproduct of hydrolyzed DOX. With decreasing pH to below 6.5, the peak intensities of released DOX-modified α-CDs increased, and the peak corresponding to DOX-HPR disappeared after incubation at pH 5.0 for 24 h (Figure 5B). The release profiles of DOX-modified α-CDs determined from the peak area ratio are shown in Figure 5C. This result clearly suggests that DOX-HPR maintained its interlocked structure under neutral to mildly alkaline pH conditions, whereas DOX-modified α-CDs were readily released under acidic conditions corresponding to the pH in lysosomes (pH 5.0).

Next, we assessed the release profiles of DOX-modified α-CDs with time at pH 7.4 and 5.0 (Figure 5D). The peak intensities for the DOX-modified α-CDs increased slightly with increasing incubation time at pH 7.4, and their release rate was only 17.5% after 24 h (Figure 5E). In contrast, the release rate of DOX-modified α-CDs gradually increased with incubation time at pH 5.0 and reached 98.4% after 24 h (Figure 5D,E). In our previous study, we investigated the dissociation profiles of HPR under varying pH [25,29]. The dissociation profiles of DOX-HPR (the release profiles of DOX-modified α-CDs) were comparable to those of HPR, indicating that the modification of DOX onto HPR did not affect its dissociation properties. Consequently, DOX-HPR released DOX-modified α-CDs completely within 24 h via dissociation under acidic pH conditions, whereas it is stable at physiological pH.

### 2.3. Cell Viability and Intracellular Localization of DOX-HPR

**Figure 6:**
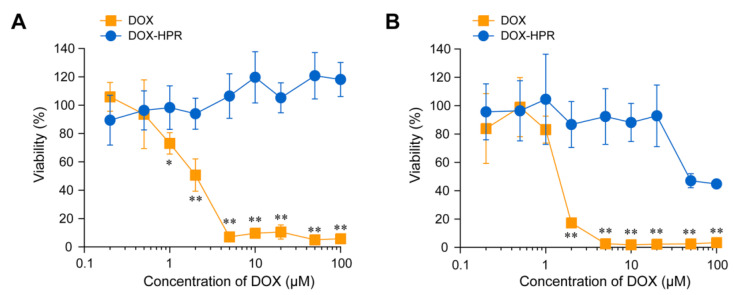
(**A**) Viability of colon-26 cells treated with DOX and DOX-HPR for (**A**) 24 h and (**B**) 48 h. Data are expressed as mean ± standard deviation (n = 6). * *p* < 0.05, ** *p* < 0.0001 vs. untreated cells (100% cell viability).

DOX mediates its cytotoxicity by intercalation into double-stranded DNA and inhibition of topoisomerase II, which plays a crucial role in DNA replication [4,5]. To assess the cytotoxic effect of DOX-HPR, colon-26 cells were treated with DOX-HPR and DOX for 24 and 48 h. After treatment for 24 h, DOX decreased the cell viability in a concentration-dependent manner, and half-maximal inhibitory concentration (IC_50_) was determined to be 1.2 ± 0.7 μM (Figure 6A). On the other hand, DOX-HPR did not show cytotoxicity even at a DOX concentration of 100 μM. The cytotoxicity of DOX after 48 h of treatment was the same as that after 24 h, and the IC_50_ was 1.3 ± 0.4 μM (Figure 6B). The viability of DOX-HPR-treated cells decreased significantly at a DOX concentration of > 50 μM. Although a significant decrease in cell viability was observed for DOX-HPR, the cytotoxic effect was weaker compared to that of DOX. It is postulated that the conjugation of DOX with α-CDs decreases the intrinsic activity of DOX.

**Figure 7:**
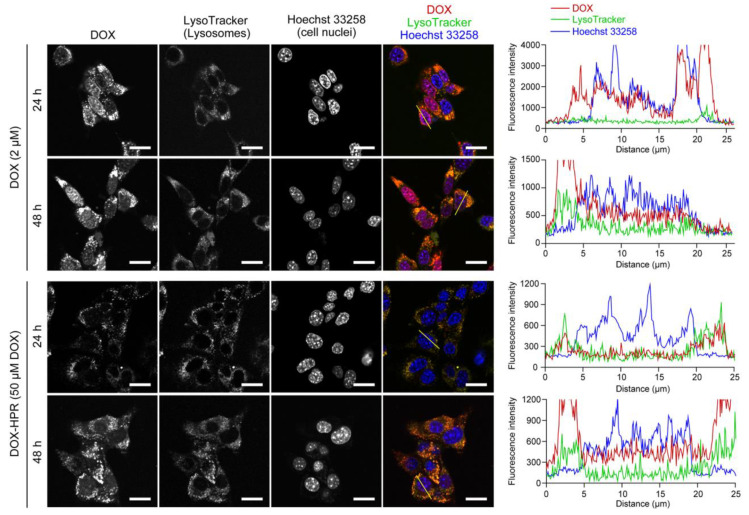
CLSM images of colon-26 cells treated with DOX (2 μM) and DOX-HPR (50 μM DOX) for 24 h and 48 h (scale bars: 20 μm). Cellular nuclei and lysosomes were stained with Hoechst 33258 and LysoTracker Green, respectively. The histograms depict the fluorescence intensity line profiles for the yellow lines in the merged images. Red, green, and blue lines denote DOX, LysoTracker Green, and Hoechst 33258, respectively.

To confirm these hypotheses, the intracellular localization of DOX was observed using CLSM after 24 and 48 h of the treatment (Figure 7). Cellular nuclei and lysosomes were stained with Hoechst 33258 and LysoTracker Green, respectively, to analyze the localization of DOX. Due to the low fluorescence intensity of DOX-HPR (Figure 4A), it was tested at a DOX concentration of 50 μM. When the cells were treated with free DOX, it accumulated in cellular nuclei due to the intercalation into DNA was clearly observed after 24 and 48 h of treatment. The fluorescence intensity line profiles for DOX were observed at the cellular nucleus fractions, confirming that DOX was localized in the cellular nuclei. In contrast, DOX-HPR did not accumulate in cellular nuclei and was predominantly localized in lysosomes after 24 h, suggesting that DOX-HPR was internalized into the cells through endocytosis. The fluorescence intensity line profiles for DOX-HPR indicated that the fluorescence derived from DOX-HPR was not observed in the cellular nuclei. However, after 48 h, slight fluorescence of DOX-HPR was observed in the cellular nuclei, which was also confirmed in the fluorescence intensity line profiles.

The CLSM results suggest that the nuclear localization of DOX-modified α-CDs was slower compared to that of free DOX. There are several possible reasons for this, such as differences in their cellular uptake pathways, release of DOX-modified α-CDs in cells, and insufficient nuclear entry of DOX-modified α-CDs. In our previous study, intracellular uptake and the dissociation of HPR was observed in cells even after 1 h of the treatment [29], suggesting that cellular entry and subsequent release were not major problems. After the release of DOX-modified α-CDs in acidic lysosomes, the DOX-modified α-CDs should diffuse into the cytoplasm to exert their anticancer effects [4,5,6]. However, the modification of DOX with α-CDs changes the properties of DOX, resulting in inhibiting the diffusion into cytoplasm, nuclear entry, and intercalation into DNA because of the hydrophilic nature of α-CDs or steric hindrance due to conjugation with α-CDs [4,5,6]. Therefore, we considered that a period of time is necessary for DOX-HPR to reach the cellular nuclei; thus, cell death was observed at 48 h.

Overall, this study demonstrates that acid-degradable PRXs act as a carrier for anticancer drugs, releasing threaded drug-modified α-CDs to elicit cytotoxicity. For improving the cytotoxic effects of the PRX–drug conjugates, DOX-HPR should be designed to release unmodified DOX from DOX-HPR via the cleavage of the linkage between DOX and α-CD under intracellular conditions. Alternatively, it is necessary to select other anticancer drugs that are not deactivated by conjugation with α-CD.

## 3. Materials and Methods

### 3.1. Instrumentation

^1^H NMR spectra were recorded on a Bruker Avance III 400 MHz spectrometer (Bruker BioSpin, Rheinstetten, Germany) in dimethyl sulfoxide (DMSO)-*d*_6_ and D_2_O at 25 °C. Chemical shifts in the ^1^H NMR spectra were referenced using DMSO (2.5 ppm) and HDO (4.65 ppm). SEC was performed using a Prominence-i LC-2030 Plus system (Shimadzu, Kyoto, Japan) equipped with an RID-20A refractive index and UV detectors and a combination of TSKgel α-4000 and α-2500 columns (300 mm length, 7.8 mm internal diameter; Tosoh, Tokyo, Japan). The sample solutions were injected into the system and eluted with DMSO containing 10 mM LiBr at a flow rate of 0.35 mL/min at 60 °C. DLS measurements were performed on a Zetasizer Nano ZS (Malvern Instruments, Malvern, UK) with a 4 mW He–Ne laser (633 nm) at a detection angle of 173°. Fluorescence spectra were recorded on an FP-8500 (Jasco, Tokyo, Japan) at an excitation wavelength of 480 nm.

### 3.2. Synthesis of α-CD-/PEG-Based Acid-Degradable PRXs

PEG-NH_2_ (*M*_n_ = 19,800) was synthesized according to a previously reported method [41]. PEG-NH_2_ (4.0 g, 202 μmol) and α-CD (9.7 g, 9.97 mmol; Ensuiko Sugar Refining, Tokyo, Japan) were dissolved in distilled water (56 mL), and the solution was stirred for 24 h at room temperature. The precipitate was collected by centrifugation (5000 rpm, 10 min) and freeze-dried to obtain pseudopolyrotaxane (6.6 g). Then, Trt-Gly-OH (1.25 g, 3.94 mmol; Tokyo Chemical Industry, Tokyo, Japan), 4-(4,6-dimethoxy-1,3,5-triazin-2-yl)-4-methylmorpholinium chloride (DMT-MM; 1.09 g, 3.94 mmol; Fujifilm Wako Pure Chemical, Osaka, Japan) were dissolved in a mixture of methanol (MeOH) and acetonitrile (MeCN) (55 mL; MeOH:MeCN = 1:4, *v*:*v*), and the solution was combined with the pseudopolyrotaxane. The mixture was stirred for 24 h at room temperature. After the reaction, the precipitate was collected by centrifugation, dissolved in DMSO, reprecipitated in distilled water, and collected by centrifugation. The reprecipitation process was repeated until the free α-CD and unreacted reagents were completely removed. The recovered precipitate was freeze-dried to yield acid-degradable PRX (2.46 g, 15.7% yield based on recovered PEG mol%). The ^1^H NMR spectra of PRX in NaOD/D_2_O were obtained, and the number of threaded α-CDs in PRXs was determined from the integration of the peaks at 3.2–3.7 ppm (m, -O-C*H_2_*-C*H_2_*- of PEG axis, H_2_, H_3_, H_4_, H_5_, and H_6_ protons of α-CD) and 4.88 ppm (anomeric proton of α-CD). The number-averaged molecular weight (*M*_n_) of PRX was calculated based on the number of threaded α-CDs in the PRXs. ^1^H NMR (400 MHz, NaOD/D_2_O) δ = 3.2–4.1 (-O-C*H_2_*-C*H_2_*- of PEG axis, H_2_, H_3_, H_4_, H_5_, and H_6_ protons of α-CD), 4.88 (m, anomeric proton of α-CD), 7.18 (t, Trt group), 7.25 (t, Trt group), and 7.33 (d, Trt group).

### 3.3. Synthesis of 2-(2-Hydroxyethoxy)ethoxy Carbamate-Modified PRX (HPR)

PRX (1.0 g, 12.9 μmol) and 1,1′-carbonyldiimidazole (CDI; 1.14 g, 7.03 mmol; Merck KGaA, Darmstadt, Germany) were dissolved in dehydrated DMSO (50 mL; Fujifilm Wako Pure Chemical), and the solution was stirred for 24 h at room temperature under a nitrogen atmosphere. Then, 2-(2-aminoethoxy)ethanol (7.0 mL, 70.6 mmol; Tokyo Chemical Industry) was added to the solution, and the reaction mixture was stirred for an additional 24 h at room temperature under a nitrogen atmosphere. After the reaction, the product was purified by dialysis against water for three days (Spectra/Por 4, Repligen, Waltham, MA, USA; molecular weight cutoff of 12,000–14,000) at 4 °C. The recovered solution was freeze-dried to yield HPR (958 mg, 78.6% yield). The ^1^H NMR spectrum of HPR in D_2_O was measured, and the number of modified HEE groups on PRXs was obtained from the integration of the peaks at 3.20 ppm (-NH-C*H_2_*-CH_2_-O-CH_2_-CH_2_-OH of the HEE group) and 4.96 ppm (anomeric proton of β-CD). The *M*_n_ of HPR was calculated based on the number of threaded α-CDs and modified HEE groups. ^1^H NMR (400 MHz, NaOD/D_2_O) δ = 3.20 ppm (-NH-C*H_2_*-CH_2_-O-CH_2_-CH_2_-OH of the HEE group), 3.33–4.56 (-O-C*H_2_*-C*H_2_*- of PEG axis, H_2_, H_3_, H_4_, H_5_, and H_6_ protons of α-CD, -NH-CH_2_-C*H_2_*-O-C*H_2_*-C*H_2_*-OH of the HEE group), 4.96 (anomeric proton of α-CD), 7.18 (t, Trt group), 7.25 (t, Trt group), and 7.33 (d, Trt group).

### 3.4. Synthesis of DOX-Conjugated HPR (DOX-HPR)

HPR (120 mg, 1.27 μmol) and CDI (5.8 mg, 35.8 μmol) were dissolved in dehydrated DMSO (6 mL), and the solution was stirred for 24 h at room temperature under a nitrogen atmosphere. Then, DOX hydrochloride (20.7 mg, 35.7 μmol; LC Laboratories, Woburn, MA, USA) and triethylamine (5.98 μL, 43.1 μmol; Tokyo Chemical Industry) dissolved in dehydrated DMSO (4 mL) were added to the reaction mixture, and the solution was stirred for an additional 24 h at room temperature under a nitrogen atmosphere. After the reaction, the product was purified using a gel filtration column (Sephadex LH-20; Cytiva, Marlborough, MA, USA) and DMF as the eluent. The product was further purified by dialysis against phosphate buffer (10 mM NaH_2_PO_4_–Na_2_HPO_4_, pH = 7.4) for three days (molecular weight cutoff of 12,000–14,000) at 4 °C. The resulting solution was desalted by passing through a gel filtration column (Sephadex G-25; Cytiva). The recovered solution was freeze-dried to yield DOX-conjugated HPR (DOX-HPR; 112 mg, 89.0% yield). The ^1^H NMR spectrum of DOX-HPR in DMSO-*d*_6_ was obtained, and the number of modified DOX on HPR was determined from the integration of the peaks at 7.97 ppm (DOX) and 4.80 ppm (anomeric proton of α-CD). The *M*_n_ of DOX-HPR was calculated based on the number of threaded α-CDs, modified HEE groups, and modified DOX. ^1^H NMR (400 MHz, DMSO-*d*_6_) δ = 3.13 ppm (-NH-C*H_2_*-CH_2_-O-CH_2_-CH_2_-OH of the HEE group), 3.18–4.28 (-O-C*H_2_*-C*H_2_*- of PEG axis, H_2_, H_3_, H_4_, H_5_, and H_6_ protons of α-CD, -NH-CH_2_-C*H_2_*-O-C*H_2_*-C*H_2_*-OH of the HEE group), 4.60 (O_6_H proton of α-CD), 4.80 (anomeric proton of α-CD), 5.80 (O_2_H and O_3_H protons of α-CD) 7.08 (-O-C(=O)-N*H*- of the HEE group) 7.20 (t, Trt group), 7.30 (t, Trt group), and 7.40 (d, Trt group), 7.63 (DOX), 7.71 (DOX), 7.97 (DOX).

### 3.5. Release of DOX-Modified α-CD from DOX-HPR

DOX-HPR was dissolved in PBS (10 mM NaH_2_PO_4_–Na_2_HPO_4_, 150 mM NaCl, pH 6.0, 6.5, 7.4, and 8.0) or acetate-buffered saline (10 mM CH_3_COOH–CH_3_COONa, 150 mM NaCl, pH 5.0 and 5.5) at a concentration of 10 mg/mL. The solutions were incubated at 37 °C for the prescribed time. The solutions (200 μL) were neutralized with phosphate buffer (100 mM NaH_2_PO_4_–Na_2_HPO_4_, pH 7.4; 200 μL) and were freeze-dried. The resulting powder was dissolved in DMSO (1 mL). SEC measurements were performed as described above, and the release profiles of DOX-conjugated α-CD were calculated from the peak areas.

### 3.6. Cell Viability

Colon-26 cells, derived from mouse colon cancer, were obtained from Riken BioResource Research Center (Ibaraki, Japan). The cells were cultured in Dulbecco’s modified Eagle’s medium (Fujifilm Wako Pure Chemical) supplemented with 10% heat-inactivated fetal bovine serum (Gibco, Grand Island, NY, USA), 100 units/mL penicillin (Fujifilm Wako Pure Chemical), and 100 µg/mL streptomycin (Fujifilm Wako Pure Chemical) in 5% CO_2_ at 37 °C. Colon-26 cells were plated in a 96-well plate at a density of 2.5×10^3^ cells/well and incubated overnight. The cells were then cultured in treatment medium containing DOX or DOX-HPR (0.1–100 μM DOX) for 24 or 48 h at 37 °C. To determine cell viability, the medium was replaced with fresh medium containing 10% Cell Counting Kit 8 reagent (Dojindo Laboratories, Kumamoto, Japan), and the cells were incubated for 1 h at 37 °C. Absorbance was measured at 450 nm using a Multiskan FC microplate reader (Thermo Fisher Scientific, Waltham, MA, USA). Cell viability was determined by comparing the results with the absorbance of untreated cells.

### 3.7. Intracellular Distribution Analysis

Colon-26 cells were plated in a 35 mm glass-bottom dish (diameter of glass area 12 mm; Iwaki, Tokyo, Japan) at a density of 1.0 × 10^4^ cells/dish and incubated overnight. The cells were then cultured in treatment medium containing DOX (2 μM) or DOX-HPR (50 μM DOX) for 24 or 48 h at 37 °C. The cells were stained with 200 nM LysoTracker Green (Thermo Fisher Scientific) at 37 °C for 60 min, followed by staining with 1 μg/mL Hoechst 33258 (Dojindo Laboratories) at 37 °C for 10 min. Confocal laser scanning microscopy (CLSM) was performed using FluoView FV10i (Olympus, Tokyo, Japan). The excitation and emission wavelengths for Hoechst 33258, LysoTracker Green, and DOX were 405 and 455 nm, 473 and 510 nm, and 473 and 610 nm, respectively.

### 3.8. Statistical Analysis

Statistical analyses were performed using OriginPro 2021 software (OriginLab, Northampton, MA, USA). Statistical differences among more than three groups were determined using one-way analysis of variance (ANOVA) followed by the Tukey–Kramer multiple comparison test. Statistical significance was set at *p* < 0.05.

## 4. Conclusions

In this study, DOX-HPR was designed for releasing DOX-modified α-CDs through the dissociation of acid-degradable PRXs under acidic conditions, such as those in lysosomes. DOX-modified α-CDs were released from DOX-HPR at pH 5.0, whereas DOX-HPR retained its interlocked structure at pH 7.4. Therefore, the selective release of DOX-modified α-CDs from DOX-HPR under acidic conditions was accomplished by conjugation of DOX with acid-degradable PRXs. Additionally, the conjugation of DOX onto acid-degradable PRXs did not affect their dissociation profiles. Although the cytotoxicity of DOX-HPR was attenuated compared to free DOX, significant cell death was observed in DOX-HPR-treated colon-26 cells after 48 h of treatment. CLSM observations revealed that the nuclear localization of DOX-modified α-CDs was observed at 48 h, whereas free DOX accumulated in cellular nuclei after 24 h. Therefore, the conjugation of DOX with α-CDs resulted in a delay in nuclear localization compared to that of free DOX. Consequently, this study demonstrated that acid-degradable PRXs act as carriers for DOX and are capable of releasing threaded DOX-modified α-CDs under acidic conditions in lysosomes and eliciting cytotoxicity. Although fine-tuning the molecular design is necessary for improving the anticancer activity, the concept of PRX–drug conjugates would contribute to the development of DDS for cancer chemotherapy.

## Figures and Tables

**Table 1:** Characterization of PRXs used in this study.

Code	Number of Threading α-CDs	Number of Modified HEE Groups	Number of Conjugated DOX	*M*_n_
PRX	58.8	-	-	77,600
HPR	58.8	129	-	94,600
DOX-HPR	58.8	129	8.1	99,200

## Data Availability

The data in this work are available from the corresponding author upon reasonable request.
